# Frequency-Aware Diffusion Model for Multi-Modal MRI Image Synthesis

**DOI:** 10.3390/jimaging11050152

**Published:** 2025-05-11

**Authors:** Mingfeng Jiang, Peihang Jia, Xin Huang, Zihan Yuan, Dongsheng Ruan, Feng Liu, Ling Xia

**Affiliations:** 1 School of Computer Science and Technology, Zhejiang Sci-Tech University, Hangzhou 310018, China; 202220602022@mails.zstu.edu.cn (P.J.); 2023110602006@mails.zstu.edu.cn (Z.Y.); ruandongsheng@zstu.edu.cn (D.R.); 2 School of Electrical Engineering and Computer Science, The University of Queensland, St. Lucia, Brisbane, QLD 4072, Australia; feng@eecs.uq.edu.au; 3 Department of Biomedical Engineering, Zhejiang University, Hangzhou 310027, China; xialing@zju.edu.cn

**Keywords:** multi-modal MRI, diffusion model, wavelet transform, MRI image synthesis

## Abstract

Magnetic Resonance Imaging (MRI) is a widely used, non-invasive imaging technology that plays a critical role in clinical diagnostics. Multi-modal MRI, which combines images from different modalities, enhances diagnostic accuracy by offering comprehensive tissue characterization. Meanwhile, multi-modal MRI enhances downstream tasks, like brain tumor segmentation and image reconstruction, by providing richer features. While recent advances in diffusion models (DMs) show potential for high-quality image translation, existing methods still struggle to preserve fine structural details and ensure accurate image synthesis in medical imaging. To address these challenges, we propose a Frequency-Aware Diffusion Model (FADM) for generating high-quality target modality MRI images from source modality images. The FADM incorporates a discrete wavelet transform within the diffusion model framework to extract both low- and high-frequency information from MRI images, enhancing the capture of tissue structural and textural features. Additionally, a wavelet downsampling layer and supervision module are incorporated to improve frequency awareness and optimize high-frequency detail extraction. Experimental results on the BraTS 2021 dataset and a 1.5T–3T MRI dataset demonstrate that the FADM outperforms existing generative models, particularly in preserving intricate brain structures and tumor regions while generating high-quality MRI images.

## 1. Introduction

In medical image analysis, multi-modal MRI provides diverse tissue contrasts and complementary information essential for a comprehensive assessment of human anatomical structures and functions [1]. By combining images from various modalities, it overcomes the limitations of single imaging techniques, thus enhancing diagnostic accuracy [2]. For example, in brain tumor diagnosis, various MRI techniques, like T1-weighted, T2-weighted, and FLAIR, are commonly used to generate images with different contrasts [3]. Moreover, MRI systems with different magnetic field strengths, like 1.5T and 3T, produce images of varying quality, with higher field strengths generally yielding better quality [4]. However, due to economic constraints, scan time limitations, and artifacts, obtaining complete multi-modal MRI images in clinical practice faces significant challenges [5,6,7].

High-quality multi-modal MRI is often the foundation for downstream tasks, and the absence of certain modalities may result in the loss of critical pathological information, thereby affecting the accuracy and reliability of analysis. For example, in brain tumor segmentation, multi-modal MRI provides comprehensive lesion information across different modalities, which helps improve segmentation accuracy and robustness. Moreover, many deep learning and machine learning algorithms rely on multi-modal data as input, and missing modalities can significantly degrade model performance. Generating high-quality images can effectively address this issue and ensure optimal model performance.

Medical image translation offers a powerful solution for transferring images from one domain to another by generating missing target modalities under the guidance of an acquired source modality [8,9,10]. Generative Adversarial Networks (GANs) have been widely applied in medical imaging [11,12,13]. However, these networks still face challenges, such as low mapping reliability and mode collapse, limiting their performance in MRI translation tasks [14]. Denoising Diffusion Probabilistic Models (DDPMs) [15] can be trained to denoise samples corrupted by varying degrees of Gaussian noise and produce high-quality images.

**Figure 1:**
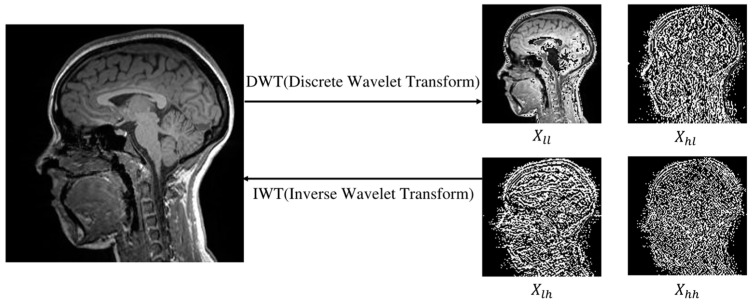
Visualization of the low-frequency component ($X_{ll}$) and high-frequency components ($X_{hl}$, $X_{lh}$, $X_{hh}$) decomposed from MRI images using a wavelet transform. The high-frequency component represents the edge part, and the low-frequency component represents the body part, so that they can be used as the diffusion conditions, capturing the main information of MRI images while saving time.

Recently, diffusion models have become potential alternatives to GANs due to their powerful generation capabilities. Several conditional diffusion models [16,17,18] have shown promising results in image-to-image translation tasks. However, these models typically start denoising from Gaussian noise, which lacks structural information from the target data distribution, presenting challenges for source-to-target MRI translation. Recent studies have introduced the I^2^SB [19] framework, a powerful class of conditional diffusion models using a novel Schrödinger bridge computation paradigm to construct a diffusion bridge between source and target image distributions. I^2^SB has achieved great results in natural image restoration but faces limitations in multi-modal MRI generation due to differences between MRI and natural images. Additionally, diffusion models often require significant computational resources, limiting their clinical applicability. In contrast, the discrete wavelet transform [20] can effectively extract low- and high-frequency components from MRI images with limited computational resources, facilitating the extraction of critical features and enabling precise capture of tissue structure information (as shown in Figure 1). By adaptively handling these components, it enables faster processing while maintaining good generation quality.

**Figure 2:**
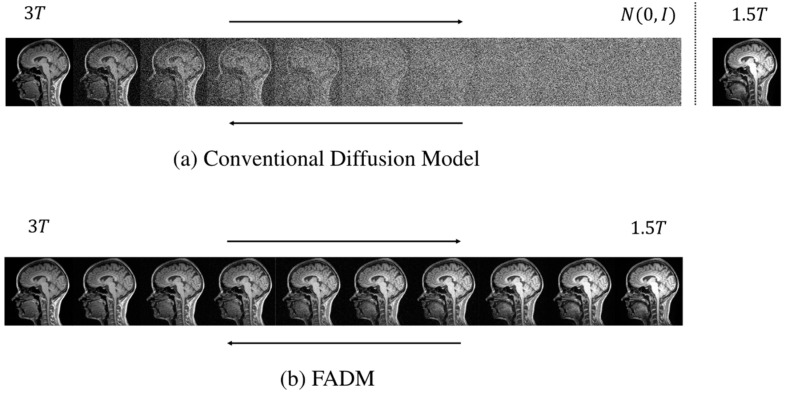
Comparison of (**a**) the conventional diffusion model and (**b**) the FADM in generating target 3T images from source 1.5T images. Unlike conventional diffusion models, the FADM adopts I^2^SB’s training strategy, focusing not on generating images from random noise but on directly learning diffusion bridges between degraded and clean distributions. This strategy yields more interpretable generation results, enhancing its applicability for image restoration.

To address these challenges, we propose a novel Frequency-Aware Diffusion Model (FADM) to enhance the quality and efficiency of MRI image generation. Unlike conventional diffusion models, the FADM establishes a diffusion bridge between source and target image distributions, progressively transforming a source image into the target image (as illustrated in Figure 2).

By integrating a wavelet transform [20], the FADM decomposes images into low-frequency and high-frequency components, effectively capturing structural and textural features of tissues during diffusion. This reduces model parameters and accelerates training, lowering computational costs. We also use a Latent Diffusion Model (LDM) [21] as a pre-trained model to generate source-conditioned images with more target-specific information. These images serve as additional inputs for the FADM, improving sampling precision. We further enhance the model by integrating a wavelet downsampling layer within the network, enhancing the frequency-aware feature representations to better capture tissue structure details. Additionally, we introduce a high-frequency feature supervision module to augment the network’s capability in extracting fine high-frequency details from MRI images.

The FADM is a source-to-target modality diffusion model designed for multi-modal MRI image generation. We test the FADM across various MRI source–target modality scenarios and extensively evaluate its utility in multi-contrast MRI conversion. Both quantitative and qualitative results show that the FADM outperforms GANs [22,23] and conventional diffusion models [18,19] in generating high-quality MRI images. Due to its reduced computational cost and enhanced generation quality, the FADM demonstrates promising potential for real-time applications in clinical environments. The main contributions are as follows:

(1) We propose a novel FADM framework that maps a provided source MRI image to target images. By integrating a wavelet transform into the diffusion process and feature space, the FADM accelerates diffusion models while improving the visual quality of the generated results.
(2) We incorporate a wavelet downsampling layer into the network, enabling the mapping of original signals or feature maps at each layer to subsequent feature dimensions in the feature space. This enhances the frequency-aware embedding of features, improving the model’s ability to capture detailed tissue structures.
(3) We employ a high-frequency feature supervision module during the network’s upsampling phase. This module uses a cross-attention mechanism to extract high-frequency information from conditional images, enhancing the network’s ability to extract high-frequency features and improve the prediction of fine details in MRI images.
(4) Comprehensive experiments conducted on the BraTS 2021 dataset and the 1.5T–3T MRI dataset verify that our FADM achieves state-of-the-art performance in terms of both quality and efficiency compared to competitive methods [18,19,22,23].

## 2. Related Work

### 2.1. GAN-Based Medical Image Translation

Generative Adversarial Networks (GANs) have achieved significant success in image-to-image translation tasks by conducting continuous adversarial training between a generator and a discriminator [22,23,24,25]. Isola et al. [22] first introduced the Pix2pix framework, a conditional GAN-based image-to-image translation model that learns the mapping from input to output images for domain translation. CycleGAN [23] extends the Pix2pix approach by enabling image generation even with unpaired data. GAN-based methods have been extensively applied in medical image translation tasks [11,12,13,26]. Despite the remarkable achievements of GANs in image generation, they still encounter several challenges. These include lower reliability in the mapping process during single-pass sampling and mode collapse, which results in insufficient representation diversity [14]. These issues limit the generalization capabilities of GANs in MRI translation tasks.

### 2.2. Diffusion Model-Based Image-to-Image Translation

In recent years, diffusion probabilistic models have surpassed traditional GAN-based models in image generation, achieving state-of-the-art results [15,27,28,29,30,31]. Several conditional diffusion models [16,17,18] have been widely applied to various image generation tasks. SR3 [18], for instance, upsamples low-resolution images to the target resolution, concatenates them with noise along the channel dimension, and then progressively denoises to generate high-resolution images. With modifications, SR3 can be adapted for image translation tasks where the input and output images have the same size.

Latent Diffusion Models (LDMs) [21] offer efficient pre-trained models for image generation. By adapting these pre-trained models, we can successfully generate conditional images tailored for specific modality translation tasks, significantly saving training time and resources. However, these methods usually begin by denoising from Gaussian noise to generate the target image. In this process, the source image is only used as a condition in the U-Net to guide the diffusion toward the target domain. Gaussian noise generally lacks structural information about the target data distribution. In contrast, the source images and the conditional images generated by the pre-trained models contain more relevant structural information about the target. WaveDiff [30] introduces Wavelet Diffusion Models, incorporating wavelet transforms into the diffusion process to balance generation quality and sampling speed, achieving strong results on natural image tasks.

Recent research on the Image-to-Image Schrödinger Bridge (I^2^SB) framework [19] has demonstrated excellent performance in various natural image restoration tasks by constructing a diffusion bridge between source and target image distributions. However, in the task of multi-modal MRI generation, there is a significant difference between the tissue structural details generated by I^2^SB and the real information due to the notable distinctions between MRI images and natural images in terms of texture details and information. To overcome these limitations, we develop the Frequency-Aware Diffusion Model (FADM), an innovative method that extends the I^2^SB framework. The FADM is designed to enhance the quality of multi-modal MRI image synthesis while improving training efficiency and reducing memory requirements. This advancement opens new avenues for future applications in multi-modal medical imaging processing.

## 3. Method

In this study, we denote $X_{0}$ as the target modality MRI image, $X_{T}$ as the source modality MRI image, and $X_{t}$ as the MRI image at time step *t* (*t* ranges from 0 to *T*). To improve sampling efficiency and image quality, we use the Haar wavelet transform at each time step (Figure 1). During sampling, we decompose the input MRI image into low- and high-frequency wavelet sub-bands and then combine them into a complete image. Concurrently, the condition image undergoes the wavelet transform to obtain *y*. We then input the complete image and *y* into the network model FAUnet to predict noise. To enhance the details, we incorporate a high-frequency feature supervision module to extract high-frequency information from the conditional image.

**Figure 3:**
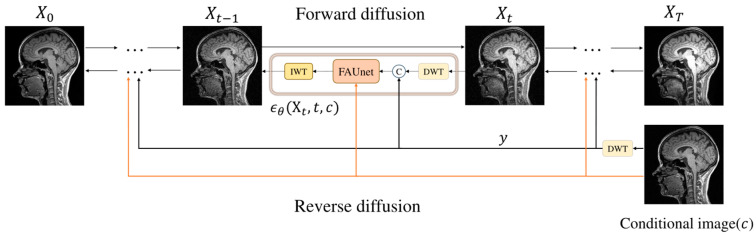
Overall framework of the FADM. $X_{0}$ represents the target modality 3T MRI image at t=0, and $X_{T}$ represents the source modality 1.5T MRI image at t=T. $\epsilon_{\theta }$ represents the network output noise. At each time step t, the input image $X_{t}$ is decomposed into four wavelet sub-bands using the discrete wavelet transform (DWT). The conditional image is also decomposed into sub-bands, denoted as y. These sub-bands (from both $X_{t}$ and the conditional image) are then fused and fed into the network to predict noise (illustrated by the black path). Concurrently, the conditional image is directly fed into the network to extract high-frequency information via the high-frequency feature supervision module (illustrated by the orange path).

The predicted noise is in the wavelet domain, so we transform the predicted noise information back to the original space using the inverse wavelet transform (IWT). This process generates the denoised sample $X_{t-1}$, and through iterative *T* steps, we obtain high-quality denoised MRI images. The overall process of the FADM is shown in Figure 3. Each sampling step occurs in the wavelet domain, where the spatial area of the wavelet sub-bands is only one-quarter of the original image. This approach significantly reduces computational complexity while preserving rich detail and structural information. This method is similar to Latent Diffusion Models (LDMs), both aiming to reduce computational load by performing diffusion in a lower-dimensional feature space. However, unlike LDMs, which require training an autoencoder for dimensionality reduction, our model only uses a wavelet transform for dimensionality reduction without the need for additional training.

### 3.1. Schrödinger Bridge Diffusion Model

The Schrödinger Bridge (SB), proposed by Erwin Schrödinger [32] in 1932, is an entropy-regularized optimal transport model. It leverages forward and backward stochastic differential equations (SDEs) to connect source and target distributions, facilitating efficient image generation. The SB model is governed by the following SDEs:

(1) $$dX_{t}=\left[f_{t}+\beta_{t}\nabla \log\Psi_{t}\left(X_{t}\right)\right]dt+\sqrt{\beta_{t}}dW_{t}$$

(2) $$dX_{t}=\left[f_{t}-\beta_{t}\nabla \log{\hat{\Psi }}_{t}\left(X_{t}\right)\right]dt+\sqrt{\beta_{t}}d{\bar{W}}_{t}$$

where $X_{t}\in R^{d}$ represents a d-dimensional stochastic process indexed by discrete time steps t∈[0,T]. $W_{t}$ and ${\bar{W}}_{t}$ denote forward and backward Wiener processes (standard Brownian motion), respectively. $X_{0}\sim p_{A}$ and $X_{T}\sim p_{B}$ are sampled from boundary distributions in two different domains. The functions $\Psi_{t}$ and ${\hat{\Psi }}_{t}$ represent time-varying energy potential fields that satisfy the following coupled partial differential equations (PDEs):

(3) $$\frac{\partial \Psi_{t}\left(x\right)}{\partial t}=-\nabla \Psi^{˕}f-\frac{1}{2}\beta ∆\Psi$$

(4) $$\frac{\partial {\hat{\Psi }}_{t}\left(x\right)}{\partial t}=-\nabla \cdot \left(\hat{\Psi }f\right)+\frac{1}{2}\beta ∆\hat{\Psi }$$

(5) $$s.t.\;\Psi_{0}\left(x\right){\hat{\Psi }}_{0}\left(x\right)=p_{A}\left(x\right),\Psi_{T}\left(x\right){\hat{\Psi }}_{T}\left(x\right)=p_{B}\left(x\right)$$

These PDEs can be viewed as generalizations of the Fokker–Planck equations, characterizing the evolution of probability densities associated with the forward and backward processes, where drift and diffusion components are governed by $f_{t}$ and $\beta_{t}$, respectively.

Through these forward and backward SDEs and coupled PDEs, the SB model constructs a smooth probabilistic path from the source to the target distribution, making it effective for cross-modal MRI image conversion. However, practical applications of the SB model involve complex stochastic process simulations and resource-intensive iterative procedures [33], since solving the coupled PDE system requires careful numerical approximation and iterative refinement of the potentials $\Psi_{t}$ and ${\hat{\Psi }}_{t}$. To overcome this, we draw on the concepts from I^2^SB [19] and transform the SB model into the following SDEs:

(6) $$dX_{t}=f_{t}(X_{t})dt+\sqrt{\beta_{t}}dW_{t}$$

(7) $$dX_{t}=f_{t}(X_{t})dt+\sqrt{\beta_{t}}d{\bar{W}}_{t}$$

where the drift term $f_{t}(X_{t})$ is shared between the forward and backward processes, and the randomness is captured through distinct noise realizations.

This form adheres to the basic structure of score-based generative models (SGMs) [34], where $\nabla \log\Psi_{t}\left(X_{t}\right)$ and $\;\nabla \log{\hat{\Psi }}_{t}\left(X_{t}\right)$ are the score functions of the above SDEs. By transforming the dynamics into a symmetric form, it becomes feasible to learn the forward and backward score functions separately without explicitly solving the potentials, thereby greatly reducing computational complexity. This transformation simplifies the model, making it more suitable for practical applications.

We explore practical strategies for applying the above model to generate target modality MRI images from a source modality MRI image. During training, paired information is obtained, i.e., $p\left(X_{0},X_{T}\right)=p_{A}\left(X_{0}\right)p_{B}\left(X_{T}|X_{0}\right)$, where $p_{A}$ and $p_{B}$ correspond to the data distributions of target and source modality MRI images, respectively. For instance, $X_{0}$ is sampled from 3T MRI image data, and ${\;X}_{T}$ is sampled from 1.5T MRI image data. The assumption of access to paired samples enables the modeling of a conditional Schrödinger Bridge, where the interpolation path depends on the initial condition, allowing personalized mappings rather than purely distributional ones. This framework enables us to construct a solvable Schrödinger bridge between $X_{0}$ and $p_{B}\left(X_{T}|X_{0}\right)$. According to I^2^SB [19], given boundary paired data ${(X}_{0},X_{T})$, we can adopt the following analytical posterior distribution:

(8) $$q\left(X_{t}|X_{0},X_{T}\right)=N(X_{t};\mu_{t}{(X}_{0},X_{T}),\sum_{t}),$$

(9) $$\mu_{t}=\frac{{\bar{\sigma }}_{t}^{2}}{{\bar{\sigma }}_{t}^{2}+\sigma_{t}^{2}}X_{0}+\frac{\sigma_{t}^{2}}{{\bar{\sigma }}_{t}^{2}+\sigma_{t}^{2}}X_{T},\;\sum_{t}=\frac{{\sigma_{t}^{2}\bar{\sigma }}_{t}^{2}}{{\bar{\sigma }}_{t}^{2}+\sigma_{t}^{2}}\cdot I$$

where $\sigma_{t}^{2}:=\int_{0}^{t}\beta_{\tau }d\tau$ and ${\bar{\sigma }}_{t}^{2}:=\int_{t}^{1}\beta_{\tau }d\tau$ represent the variances accumulated from either end, respectively. The terms $\mu_{t}{(X}_{0},X_{T})$ and $\sum_{t}$ are the mean and covariance of the conditional distribution, describing the distribution at time *t*. Intuitively, the mean $\mu_{t}$ represents a weighted interpolation between $X_{0}$ and $X_{T}$. The covariance $\sum_{t}$ quantifies the uncertainty at each intermediate time point, increasing toward the midpoint and vanishing at the boundaries. This analytical posterior allows for more efficient computation, leveraging existing score networks and SGM techniques for learning and inference. We adopt the same framework as I^2^SB for the Schrödinger Bridge Diffusion Model. For detailed procedures, please refer to the literature [19].

### 3.2. Frequency-Aware Generative Network (FAUnet)

**Figure 4:**
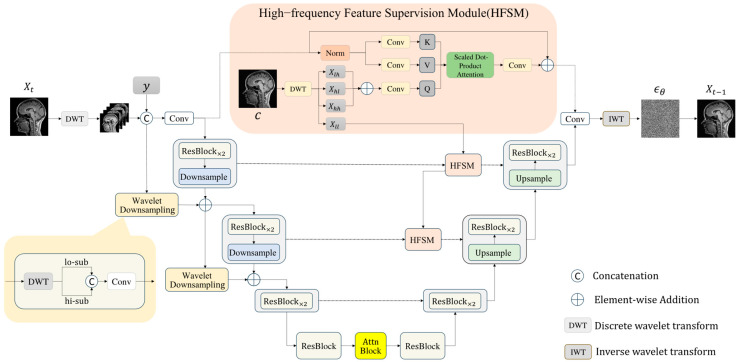
Structure of the Frequency-Aware Generative Network. The input $X_{t}$ is first decomposed into four wavelet sub-bands via the discrete wavelet transform. These sub-bands are concatenated with y and then processed through a series of modules, including downsampling, wavelet downsampling layers, high-frequency feature supervision modules, and upsampling, resulting in the final output.

This section introduces FAUnet (Frequency-Aware U-Net), a novel architecture that embeds wavelet information into the feature space to enhance structural and textural fidelity in synthesized MRI images. As illustrated in Figure 4, FAUnet extends the classic UNet [34], with several enhancements, including M downsampling and upsampling blocks, residual blocks (ResBlock), self-attention modules (Attn Block), time-step embeddings (Time Embedding), and wavelet downsampling layers. A high-frequency feature supervision module further connects encoder and decoder features at corresponding resolutions to facilitate the effective transmission of fine-grained information.

Serving as the noise prediction network within the diffusion framework, FAUnet aims to learn the score function in wavelet space, thereby guiding the denoising process and progressively reconstructing high-quality MRI images. During encoding, input images are processed through residual blocks, sequential convolutional, and wavelet downsampling layers to progressively reduce spatial resolution while capturing multi-scale representations. The bottleneck layer leverages residual and attention blocks to model global context and reinforce spatial relationships. In the decoding phase, the network gradually restores spatial resolution and predicts the noise distribution at each time step, progressively approximating the target image distribution. Meanwhile, the high-frequency feature supervision module supplies additional structural priors, improving the model’s ability to preserve high-frequency details, texture quality, and boundary sharpness.

Overall, FAUnet effectively learns the joint feature distribution in both the frequency and spatial domains, substantially improving the structural integrity and detail fidelity of the generated MRI images.

**Wavelet Downsampling Layer**. In the UNet framework [34], the original design employs stride convolution downsampling layers to incorporate the raw signal into the encoder’s various feature pyramids. In this study, we further introduce a wavelet downsampling layer to enhance frequency-based perceptive capabilities. Specifically, we first use a wavelet downsampling layer to map the input raw signal residual shortcut to the feature dimension of the first layer, which is then added to the first layer’s feature pyramid. Each subsequent layer’s feature map is decomposed into low-frequency and high-frequency sub-bands using the wavelet transform. These sub-bands are then concatenated and fed into the convolution layer for feature projection. Finally, they are added to the next layer’s feature pyramid.

The wavelet downsampling layer enables the effective separation and preservation of high-frequency and low-frequency components, enriching the network’s ability to perceive and embed frequency-specific features. By enhancing the encoder’s understanding of frequency components, this structure improves the network’s expressive capacity and downsampling operations, offering an effective frequency-aware alternative to traditional stride convolution.

**High-frequency Feature Supervision Module (HFSM)**. In the UNet framework [34], encoder features are directly connected to decoder features, facilitating effective feature integration across different levels. However, this approach is insufficient for extracting high-frequency features, which are crucial in medical image processing, especially for diagnosing and analyzing critical fine details.

To address this issue, we propose a high-frequency feature supervision module (as shown in Figure 4) to better capture and utilize high-frequency information. Specifically, we use a cross-attention mechanism for high-frequency features. First, we generate the conditional image using a pre-trained model and perform multi-level wavelet transform on the conditional image to extract high-frequency coefficients ($X_{hl}$,${\;X}_{hh}$, and $X_{lh}$). These high-frequency sub-bands are then linearly projected and summed to obtain the feature map *Q* with integrated high-frequency information:

(10) $$Q={Conv}_{1\times 1}(X_{hl}+X_{hh}+X_{lh})$$

Next, we perform different linear projections on the input feature map *M* to obtain *K* and *V* for the cross-attention mechanism:

(11) $$K={Conv}_{1\times 1}(M)$$

(12) $$V={Conv}_{1\times 1}(M)$$

Using the feature map *Q* and the projected *K* and *V*, we compute the output feature map $M^{’}$ using the following formula, where $d_{k}$ is the number of columns in matrix *Q*:

(13) $$M^{’}=Softmax\left(\frac{QK^{T}}{\sqrt{d_{k}}}\right)V$$

Finally, the output feature map $M^{’}$ is used as an additional input in the upsampling block to upsample the features based on frequency cues. This approach effectively enhances the feature recovery capability by leveraging high-frequency information.

## 4. Experiments

We evaluated the FADM on the BraTS 2021 dataset [3] and the 1.5T–3T dataset, using parameter settings based on the original I^2^SB model. The evaluation focused on comparing the quality of MRI images generated by the FADM with those produced by GAN-based methods (Pix2pix and CycleGAN) and diffusion model-based methods (SR3 and I^2^SB).

To ensure a fair comparison, we carefully tuned the hyperparameters for each baseline method by following their official implementations and parameter search recommendations provided in their original papers. For GAN-based methods, we adjusted the learning rates, generator–discriminator update frequencies, and loss function weights to achieve optimal performance on our datasets. For diffusion-based methods, such as SR3 and I2^S^B, we tuned noise schedules, the number of timesteps, and learning rates to ensure convergence and stability. Each comparative method was configured with optimal parameters, as described in its respective original paper.

To overcome the potential loss of important spatial information when slicing 3D images into 2D images, we adopted the global context compensation method. This method stacks the target 2D slice with its adjacent slices (one before and one after) to form a multi-channel input, enhancing the model’s understanding of 3D space.

### 4.1. Datasets and Implementation Details

**The 1.5T–3T Dataset.** Extensive experiments were conducted using the 1.5T–3T dataset, collected in collaboration with the Karolinska Institute in Sweden. This dataset consists of paired 3D MRI scans from 70 patients, with each pair including a 1.5T and a 3T MRI of the same patient’s brain. The scans are acquired using the MPRAGE sequence, focusing on T1-weighted imaging for high-contrast, high-SNR brain images across axial, sagittal, and coronal planes. Each 3D scan has a resolution of 1 × 1 × 1 mm^3^ and a matrix size of 256 × 256 × 176. We processed these scans by performing cross-modal registration with the deep learning-based method Voxelmorph [35], ensuring alignment between the 1.5T and 3T images. After registration, the 3D volumes were sliced into 2D images in all three planes and resampled to 256 × 256 pixels. The final dataset consisted of 27,300 pairs of 2D images, with 10,500 axial, 8400 sagittal, and 8400 coronal images. The dataset for each orientation was divided into training, validation, and test sets in an 80-10-10 ratio, ensuring subject independence across the model development phases.

**The BraTS 2021 Dataset.** To further evaluate our model’s performance in multi-modal MRI generation, we used the BraTS 2021 dataset [3]. This dataset contains four MRI modalities (T1, T1ce, T2, FLAIR). We selected T1-weighted, T2-weighted, and FLAIR-weighted brain MRI images of 100 patients for analysis and divided them into training, validation, and test sets, with 80, 10, and 10 subjects, respectively. For each subject, we selected 70 axial 2D slices containing brain tissue to assess the model’s ability to generate multi-modal MRI images. For more details on these datasets, please refer to the Appendix [3].

**Evaluation Metrics.** To evaluate the performance of the FADM, we used three metrics on the independent test set, including the Peak Signal-to-Noise Ratio (PSNR), the Structural Similarity Index Measure (SSIM), and the Frechet Inception Distance (FID). We report the mean values of these metrics, and for PSNR and SSIM, we also report their standard deviations to validate the superiority of our method in synthesis quality.

We used Python 3.9 and PyTorch 1.7.0 to program and train the proposed model on a GeForce RTX 3090 GPU (NVIDIA, Santa Clara, CA, USA) within an Ubuntu 20.04 software environment. Both input and output images were resized to 256 × 256 pixels. We used the AdamW optimizer [36], with a learning rate of 5 × 10^−5^, and set the time step *T* to 1000 and the batch size to 2. The same preprocessing steps, image resizing, and data augmentation strategies were applied consistently across all methods to eliminate any bias. We ran each model multiple times and calculated the average performance. The model with the minimum loss was saved as the optimal model.

### 4.2. Quantitative and Qualitative Comparisons

**Figure 5:**
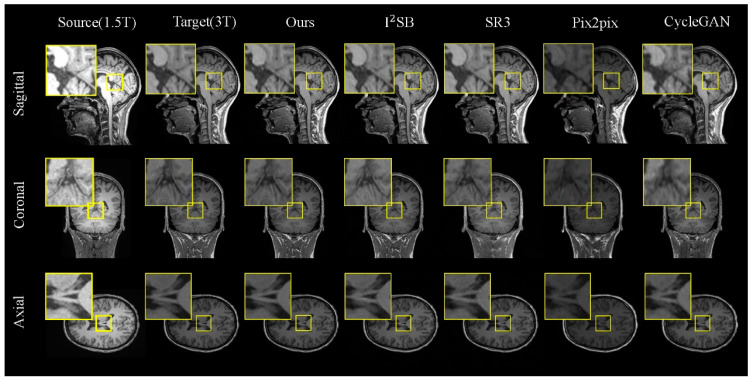
Comparisons of 2D slices (axial, coronal, and sagittal views) generated by our FADM and I^2^SB [19], SR3 [18], Pix2pix [22], and CycleGAN [23] based on our 1.5T–3T test set. Yellow boxes highlight the central region where the FADM produces more detailed and sharper textures, whereas other methods yield artifacts or blurry effects.

**Results on the 1.5T–3T Dataset.** Table 1 presents a comparative analysis of various methods on the 1.5T–3T test set, showcasing both the quantitative results and the visual outcomes for the 1.5T and 3T MRI images (as illustrated in Figure 5). For a detailed analysis, we selected local regions in three directions for magnified display (indicated by the yellow box).

In Figure 5, we observe that our model excels in generating tasks across the sagittal, coronal, and axial planes. When comparing different brain regions in the slices, although CycleGAN and Pix2pix can reproduce the overall brain morphology similar to the real images, their outputs exhibit significant discrepancies in finer details. The diffusion-based models, I^2^SB and SR3, perform better in preserving brain structural information but still fall short in capturing detailed tissue features. In contrast, the FADM excels in capturing tissue details and texture features, particularly in the sagittal and coronal plane generation tasks, where it significantly outperforms other methods. In the axial plane generation task, where the brain structures in MRI images are relatively simpler, the FADM’s results are comparable to those of I^2^SB.

The quantitative results in Table 1 further corroborate the FADM’s superiority. Our model demonstrates significant improvements in the PSNR, SSIM, and FID scores, especially in the sagittal and coronal plane generation tasks, where the FADM exhibits higher PSNR and SSIM scores and lower FID values, indicating higher image quality and better stability. These findings suggest that the FADM can be effectively utilized to generate high-quality 3T MRI images from 1.5T MRI images when the direct acquisition of 3T MRI images is challenging.

**Figure 6:**
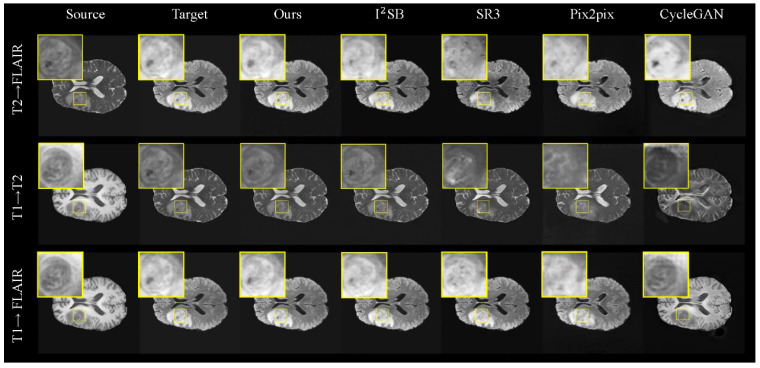
Generated MRI images for various tasks in the BraTS 2021 test set using different generation methods. The yellow boxes highlight the brain tumor region. The FADM significantly outperforms the other methods, displaying excellent texture details and structural accuracy, especially in the tumor area. The generation quality is better for the T2 modality compared to the FLAIR modality.

**Results on the BraTS 2021 Dataset.** In our evaluation on the BraTS 2021 dataset of brain tumor images, we focused on transformations between T1, T2, and FLAIR modalities (T1 → T2, T2 → T1, T1 → FLAIR, T2 → FLAIR). Table 2 presents the quantitative results for these generation tasks, and Figure 6 visualizes the generated images, highlighting the tumor region (indicated by the yellow box).

Visually, the FADM significantly outperforms the other methods. Due to the one-shot sampling nature of GANs, CycleGAN and Pix2pix produce images with significant noise and noticeable discrepancies from the real images, particularly in the T1 → T2 task, where CycleGAN’s output deviates significantly from true MRI styles. In contrast, SR3 generates brain structures closer to reality, but the tumor regions remain noisy and poorly defined. I^2^SB performs better in capturing both brain and tumor structures but still lacks the detail seen in real images. The FADM surpasses these methods by generating superior brain and tumor structures with clearer texture details, especially within the tumor regions highlighted by the yellow boundary in Figure 6.

The quantitative results in Table 2 further confirm FADM’s superior performance across various metrics. The FADM’s generated images show the highest similarity to real images, with an average PSNR that is 2.1 dB higher than I^2^SB and an average SSIM that is 2.7% higher. Additionally, the FADM achieves the lowest FID score, averaging 3.75 lower than I^2^SB. The quality of the generated images is better when the target modality is T1 or T2 compared to FLAIR. Overall, these results demonstrate our model’s exceptional fidelity and accuracy in synthesizing complex brain structures and tumor regions. Hence, our findings indicate that generating target modality MRI images from source modality MRI images is feasible and effective.

### 4.3. Ablation Study

To assess the impact of various components in our proposed method on the training process and generation quality, we conducted detailed ablation experiments on the 1.5T–3T dataset, focusing on the sagittal plane generation task. Table 3 summarizes the effects of different components on the model’s performance, highlighting the contributions of the wavelet transform, the wavelet downsampling layer, the high-frequency feature supervision module (HFSM), and the conditional image.

Experiment (a) uses the WaveDiff, which also uses wavelet transforms and follows the same approach as ours by incorporating wavelet transforms into the diffusion process. Experiment (b) calculates the baseline performance of the I^2^SB model, demonstrating the feasibility of generating multi-modal MRI images directly from source modality images, with better results than other diffusion models starting from Gaussian noise. Experiment (c) applies the wavelet transform to the input images before feeding them into the model. This significantly reduces memory usage and training time while maintaining comparable performance to the original I^2^SB model. Experiment (d) utilizes conditional images generated by a pre-trained model as additional input, greatly enhancing the generation quality and resulting in significant improvements in both PSNR and SSIM. In Experiment (e), we added the wavelet downsampling layer to enhance the perception of the frequency content, further improving PSNR (from 29.061 to 30.948) and significantly boosting SSIM. Experiment (f) incorporates a high-frequency feature supervision module (HFSM). Although this slightly increases memory usage (from 13.252 GB to 15.496 GB), it markedly improves the generation quality, with noticeable enhancements in both PSNR and SSIM.

Finally, our proposed method combines all these enhancements, achieving the best generation quality. The PSNR reaches 31.898 and SSIM achieves 0.951, demonstrating that the integration of these components effectively enhances the network’s performance and the quality of the generated MRI images. Furthermore, both our method and the WaveDiff model perform well in memory usage and training time due to the incorporation of wavelet transforms in the diffusion process. However, our method includes a wavelet downsampling layer, HFSM, and a conditional image, significantly improving image quality. Unlike WaveDiff, which starts from Gaussian noise, our method progressively maps from the source image to the target, leading to better results. These results, combined with the efficient runtime and lower computational burden of the FADM, indicate its potential to be deployed in real-world clinical environments where rapid and accurate synthesis is required.

## 5. Conclusions

In this paper, we propose a novel Frequency-Aware Diffusion Model (FADM) for multi-modal MRI image generation. By integrating a discrete wavelet transform, a wavelet downsampling layer, and a high-frequency feature supervision module, the FADM effectively captures structural details and high-frequency information, significantly enhancing image quality and synthesis efficiency. Our experiments on the 1.5T–3T and BraTS 2021 datasets show that the FADM outperforms the existing GANs and diffusion models, particularly in reproducing complex brain structures and tumor regions. Our ablation studies further validate the contribution of each component.

The FADM also demonstrates strong practical potential by enabling faster image generation, higher computational efficiency, and lower memory usage, making it suitable for real-time clinical deployment on modern hardware. Although direct validation with clinical data is limited by privacy regulations, we are actively collaborating with medical institutions to integrate the FADM into clinical workflows to support diagnosis.

In summary, the FADM offers an efficient and practical solution for generating high-quality multi-modal MRI images in clinical settings. Its lightweight design, computational efficiency, and low memory footprint make it well-suited for real-world diagnostic workflows, especially in resource-constrained environments. Our future work will focus on scaling the FADM to larger datasets and broader clinical imaging applications.

## 6. Limitations and Future Work

Despite the promising results of the proposed FADM framework, several limitations still exist. During our extensive experiments, we found that the wavelet transform decomposes image frequencies using local convolutional filters. However, due to limited neighborhood information at boundaries, convolution results may be inaccurate, especially when applied repeatedly in diffusion models, amplifying boundary artifacts. During the inverse wavelet transform, boundary regions, particularly at the tumor–tissue interface, are difficult to fully recover. Another limitation is the reliance on 2D slices extracted from 3D MRI volumes for training. While this ensures data quality and computational feasibility, it may compromise the spatial context and inter-slice consistency vital for 3D volumetric analysis. Additionally, the current framework depends on supervised learning, requiring large amounts of annotated paired data, which are costly and time-consuming to obtain in medical imaging.

In our future work, to mitigate the boundary effects caused by the wavelet transform, we will design a high-frequency feature supervision module to enhance the recovery of image details, guiding the network at each diffusion step to focus more on high-frequency structures, such as tumor boundaries. Our future research will explore alternative high-frequency enhancement mechanisms, such as attention-based residual learning or transformer-based frequency fusion, to better preserve tumor boundaries. We also plan to extend the FADM to full 3D volumetric synthesis, improving spatial consistency through architectural adaptations and memory-efficient strategies. Furthermore, self-supervised or unsupervised learning approaches will be investigated to reduce annotation costs and improve generalization to unseen domains. We also aim to expand the FADM’s application to cross-modal synthesis tasks, such as CT-to-MRI translation.

## Figures and Tables

**Table 1:** Average generation results for the 1.5T–3T test set slices (axial, coronal, and sagittal views) using different methods. The values in bold are the best ones for each evaluation matrix.

Method	Sagittal			Coronal			Axial		
	PSNR ↑	SSIM ↑	FID ↓	PSNR ↑	SSIM ↑	FID ↓	PSNR ↑	SSIM ↑	FID ↓
CycleGan	24.699 ± 2.275	0.849 ± 0.029	60.738	25.326 ± 2.361	0.857 ± 0.028	62.782	29.976 ± 1.393	0.927 ± 0.029	57.393
Pix2pix	21.374 ± 3.383	0.825 ± 0.035	71.826	23.435 ± 3.235	0.860 ± 0.042	69.745	26.486 ± 2.725	0.902 ± 0.036	54.354
SR3	28.394 ± 1.318	0.883 ± 0.016	46.033	30.640 ± 1.363	0.872 ± 0.012	41.240	31.793 ± 1.304	0.922 ± 0.017	37.246
I^2^SB	29.282 ± 1.467	0.914 ± 0.014	40.369	30.958 ± 1.334	0.926 ± 0.010	35.394	33.682 ± 1.541	0.956 ± 0.010	28.674
**Ours**	**31.897** ± **1.342**	**0.951** ± **0.008**	**33.304**	**32.208** ± **1.265**	**0.958** ± **0.008**	**30.565**	**33.854** ± **1.452**	**0.968** ± **0.007**	**26.411**

**Table 2:** Average generation quality for different tasks in the BraTS 2021 test set using various generation methods. Bold data represent the optimal results overall.

Method	T1 → T2			T2 → T1			T2 → FLAIR			T1 → FLAIR		
	PSNR ↑	SSIM ↑	FID ↓	PSNR ↑	SSIM ↑	FID ↓	PSNR ↑	SSIM ↑	FID ↓	PSNR ↑	SSIM ↑	FID ↓
CycleGan	21.776 ± 3.756	0.726 ± 0.058	69.525	19.513 ± 3.857	0.737 ± 0.059	74.079	19.207 ± 2.613	0.827 ± 0.057	78.326	19.123 ± 2.797	0.714 ± 0.031	80.386
Pix2pix	26.286 ± 2.439	0.874 ± 0.055	62.422	26.867 ± 2.490	0.825 ± 0.054	63.852	25.914 ± 3.718	0.837 ± 0.047	72.737	26.819 ± 2.245	0.855 ± 0.044	70.837
SR3	27.060 ± 1.912	0.902 ± 0.024	45.878	28.457 ± 2.404	0.873 ± 0.039	44.796	27.494 ± 2.011	0.869 ± 0.037	50.744	27.728 ± 1.204	0.874 ± 0.039	46.584
I^2^SB	30.827 ± 1.286	0.928 ± 0.015	37.473	30.423 ± 1.269	0.924 ± 0.025	40.976	29.208 ± 2.437	0.937 ± 0.024	40.361	30.145 ± 2.317	0.929 ± 0.028	39.867
**Ours**	**32.731 ± 1.186**	**0.964 ± 0.096**	**33.833**	**31.869 ± 1.485**	**0.961 ± 0.014**	**34.967**	**30.847 ± 1.338**	**0.958 ± 0.025**	**38.283**	**31.758 ± 1.478**	**0.946 ± 0.016**	**36.646**

**Table 3:** Performance metrics for the 1.5T–3T dataset in the sagittal plane: average training time per image (s), model memory load during training (GB), and average PSNR and SSIM of the generated images.

Method	Memory	Training	PSNR (↑)	SSIM (↑)
a: WaveDiff	18.652	0.308	28.743	0.896
b: I^2^SB	21.134	0.498	29.282	0.914
c: b + Wavelet Transform	13.252	0.210	29.061	0.913
d: c + Conditional Image	13.550	0.228	29.907	0.936
e: c + Wavelet Downsampling Layer	14.610	0.252	30.948	0.923
f: c + HFSM	15.496	0.265	30.639	0.939
FADM	17.506	0.315	31.898	0.951

## Data Availability

The code will be cleaned up and stored on GitHub at https://github.com/Rain-Alley/FADM (accessed on 20 April 2025), where it will be available for public viewing. The data that support the findings of this article are not publicly available due to privacy concerns. They can be requested from the author at 202220602022@mails.zstu.edu.cn.
